# Performance evaluation of geopolymer mortars containing waste ferrochrome slag and fly ash for sustainable green building

**DOI:** 10.1038/s41598-024-65552-w

**Published:** 2024-06-25

**Authors:** Şermin Koçyiğit

**Affiliations:** Department of Construction Technology, Technical Sciences Vocational School, Dicle University, Diyarbakir, Turkey

**Keywords:** Alkaline activators, Compressive strength, Geopolymer mortar, Green building design, Waste recycling, Water absorption, Environmental sciences, Engineering

## Abstract

The aim of the present study, an attempt to shed light on the use of industrial-based wastes as alkali-activated binder (AAB) material is mainly. The present novel research work, the characterization of waste ferrochrome slag (FCS) and the performance of alkali-activated mortar consisting of fly ash (FA) were investigated. The characterization of used materials were carried out using advanced microstructural analysis techniques (XRF, XRD and SEM). A total of thirty two mortars are prepared using FCS (90–60%) and FA (10–40%) with 5 M, 10 M sodium hydroxide (NaOH), Na_2_SiO_3_/NaOH (SS/SH = 1 and 2) solution. All specimens were cured in an oven at 70 °C and 100 °C for 24 h. After oven curing, the geopolymer mortars were kept in the laboratory for 28 days and thermal and mechanical tests were applied to them. The A5 mixture (SS/SH = 1 with 10%FA, 90%FCS and 5 M NaOH) was found to be optimum in terms of thermal insulation properties, making it suitable for use in sustainable construction in terms of low energy cost through exterior insulation. The C8 mixture (SS/SH = 1 with 40%FA, 60% FCS and 10 M NaOH) was found to be optimum in terms of strength and durability, making it suitable for use in sustainable construction. As a result, in this study, an optimum mixture of waste FCS and FA was obtained and geopolymer building materials that provide thermal insulation and structural performance and are resistant to external influences were produced.

## Introduction

It is important for sustainable development that energy required to meet the needs of present and future generations without harming our ecosystem are provided from renewable sources of energy and recyclable materials. Thus, new techniques are constantly being adapted and investigated to help contribute to the preservation of our world. Therefore, most of the researches and techniques are related to the construction industry, as global population growth increases the need for housing and the demand for the construction industry in parallel. Concrete is the most widely used material in the construction industry and Portland cement is the most widely used binder^1,2^. CO_2_ emission caused by cement production is about 8–10% of total CO_2_ emission^3,4^. The production of Portland cement requires high energy and causes air pollution besides carbon dioxide emissions causing climate change due to global warming^5^. Many researchers have tried to reduce total cement production by offering total or partial alternatives or by blending Portland cement with complementary cementing materials^6–11^. There are many modern building materials aiming to reduce dependency on cement by finding alternatives or improving its efficiency^12,13^. Numerous studies have focused on use of wastes and by-products in construction for years^14^. The most common by-products from industrial waste are ground blast furnace slag^15^, fly ash (FA)^16^, silica fume^17^, and cement kiln dust^18^. Those derived from natural sources include volcanic pumice powder^19^, metakaolin^20^, and palm oil fuel ash^21^. Some of these materials are used in a variety of ways and applications, while some others end up in landfills. Even when precautions are taken, heavy metals and other harmful substances in landfilled waste can leak into groundwater in time by harming the environment and the health of people living in that environment. The recycling of by products and wastes offers solution for their disposal problems and contributes to sustainability, as well^22,23^. Rapid depletion of natural resources to meet modern needs seriously threatens sustainability^24,25^. The objective of sustainability is to protect and preserving resources for the future in the process^26^. To solve this, people could use alternative construction materials instead of conventional ones. Thus far, researchers have explored numerous by-products and industrial wastes to reach that goal. An example of these wastes comes from the ferrochrome industry. Ferrochrome is a chromium and iron alloy produced by smelting chromite (containing chrome and iron oxides), in an electric arc furnace. The major industrial by-products obtained from the ferrochrome industry are the ferrochrome wastes. Examples of such wastes are the ferrochrome ash (FCA) and ferrochrome slag (FCS). Ferrochrome slag (FCS) is a by-product of that process; on the other hand, ferrochrome ash (FCA) is a by-product of the ferroalloy industry. FCA is the dust produced by gas cleaning operations, when removing harmful flue gas from smelting furnaces. The annual production of ferrochrome is in the range of 6.5–9.5 million tonnes in the world and increases with a 3% growth rate per year as an alloy. The ferrochrome manufacturing per metric tonne results in 1.0–1.6 tonnes of FCS and 0.02–0.03 tonnes of FCA by-products^27^.Given Ferrochrome slag’s mechanically competent and eco-friendly nature, people can use it in concrete as either fine or coarse aggregate^28,29^. Another example of industrial waste is fly ash, which is produced by burning coal, biomass and municipal waste to generate electricity in thermal power plants. Fly ash, a plentiful coal combustion by-product^30^, is widely considered one of the most underutilized materials globally. The fly ash global annual production exceeds 900 million tons^31^, with significant volumes generated in countries like Australia (approximately 14 million tons per year)^32^, India (169.25 million tons per year)^33^, China (580 million tons)^34^, and the USA (43.5 million tons)^35^. Unfortunately, the fly ash current rate of utilization remains low, with only 53.5% being effectively utilized worldwide^36^ and even lower in Australia at 44.34%^37^.Therefore, with the search for alternative building materials in the construction industry, the use of waste has emerged to provide both sustainable environmentally friendly buildings by emptying landfills and more economical solutions by saving energy. By determining the optimgroundum waste amounts and substituting waste for aggregate or cement in concrete production in order to provide properties close to the control specimen (that does not contain waste), alternative solutions to depleting natural resources can be found and also more economical, environmentally friendly and sustainable concrete can be produced in terms of durability. Researchers have developed a new bonding technology and environmental cement binders including geopolymer and hybrid alkali-activated concrete to solve the problem. Geopolymers can be synthesized using low energy, temperature, and cost and by utilizing diverse profuse wastes as either precursors or supplementary cementitious materials (SCM) and resolving the systematic disposal of materials such as fly ash, metakaolin, and ground granulated blast furnace slag. Geopolymer concrete (GPC) is an innovative and environmentally friendly concrete that hardens through the reaction between aluminosilicate waste materials and alkaline activating solutions rather than requiring a cement binder^38^. Precursor materials include fly ash, blast furnace slag and metakaolin, while alkaline activators consist of chemical compounds such as sodium hydroxide and sodium silicate solution. FA is a key solid waste by-product produced by coal-firing power plants. Thermal electricity stations are seeking ways to dispose of it in an economically and environmentally beneficial way, as well as CO_2_ sequestration^39,40^. Fly ash, which contains high amounts of reactive silica and alumina in its chemical composition, is widely used in the production of geopolymer composites. The reaction between ground fly ash and alkaline activators facilitates the geopolymer reaction, resulting in a dense, cross-linked polymer structure^41^. The water used in the mixing stage of geopolymers is used for processability and leaves the geopolymer during curing and drying, leaving discontinuous nano-voids. This gives the geopolymer positive properties such as light weight, thermal insulation and fire resistance^42^. Geopolymer concrete has many advantages compared to ordinary concrete. It is resistant to chemical attacks and has excellent mechanical properties such as high compressive and flexural strength. Reduced permeability, increased resistance to freeze–thaw cycles and increased fire resistance are just some of the indicators that geopolythe mer concrete outperforms traditional concrete in terms of durability. Geopolymer concrete is an attractive candidate for green construction methods due to its promise to reduce carbon emissions and energy consumption. In the literature, there are mostly no studies on the use of FCS and FA together by replacing them at different ratios. Some geopolymer studies have revealed that FCS is used as a precursor rather than a fine aggregate by bringing it to cement fineness for use in the AAB system. In this study, FCS was used both as a precursor and fine aggregate. In addition, any study has not reported the effect of different ratios of FCS and FA activated using NaOH and Na_2_SiO_3_ on thermal conductivity, mechanics and curing techniques. This study provides the novelty of using FA and FCS activated using NaOH and Na_2_SiO_3_ solutions to create an AAB system cured at different curing temperatures. The major importance of the present study is to enhance the methods of producing eco-friendly buildings by utilizing industrial wastes (FCS, FA) in obtaining zero cement binder material that will serve as a sustainable source of construction material for infrastructure development towards safe and sustainable development goals. Therefore, in the present study, the thermal and mechanical properties of geopolymer-bonded mortars produced using waste FCS and FA were investigated as an alternative to cement-bonded mortars to reduce environmental pollution caused by both cement use and industrial wastes.

## Materials and method

### Materials

#### Ferrochrome slag

Ferrochrome is an alloy obtained from chromite ore containing iron and chromium and is mostly used for production of stainless steel. Ferrochrome slag (FCS) is the waste material produced during the production of iron and steel by processing chromite ore in electric-arc furnaces in ferrochrome production facilities. FCS is produced by melting rocks and large rock rubble aggregates containing metal materials from the mine in blast furnaces at 1500–1700 °C and separating the ore and slag. The FCS, which is the subject of the study, was obtained from the Elâzığ ferrochrome factory and as 1 kg of slag waste is generated for every 3 kg of ferrochrome during the production of approximately 150.000 tons of ferrochrome per year in this factory, the amount of waste slag in 1 year is 50.000 tons. This slag contains 3–5% Cr_2_O_2_^43^.

#### Fly ash

Fly ash is an industrial waste produced by capturing micron-sized particles released from burning coal to generate electricity in thermal power plants and mixed into the atmosphere as dust, captured by electrostatic precipitator filters or filter bags in thermal power plant chimneys and stacked in fly ash silos^44^. Since this unclassified ash cannot be used in cement production, serious storage problems arise^45^. Waste fly ash was obtained from İsken-Sugözü Power Plant. Fly ash, which was ground to cement fineness and micron size and classified as F class (low calcareous) according to ASTM C618 standard due to SiO_2_ + Al_2_O_3_ + Fe_2_O_3_ value above 70% and CaO content less than 10%, was used as binder in the study.

#### Alkaline activators

NaOH is a white material usually found in the form of lumps, granules or flakes^46^. It is a material that dissolves very quickly due to its high solubility levels in water and alkalis and is frequently used in geopolymer mixtures. NaOH has a density of 2.1 g/cm^3^, a pH of 14 and 98% purity. Na_2_SiO_3_ is produced as a result of calcification of sodium carbonate (Na_2_CO_3_) and quartz (SiO_2_) at temperatures between 1400 and 1500 °C through a process that releases large amounts of carbon dioxide as a by-product^47^. Sodium silicate, also called as glass water, is an adhesive, viscous, colorless and liquid chemical product with the formula Na_2_SiO_3_ and has a wide range of applications. Na_2_SiO_3_ density is 1.38 gr/cm^3^ and it contains 8.9% Na_2_O, 28.7% SiO_2_ and 64.8% H_2_O.

### Characterisation of raw materials

#### Partical size, XRF, XRD and SEM analysis

**Figure 1:**
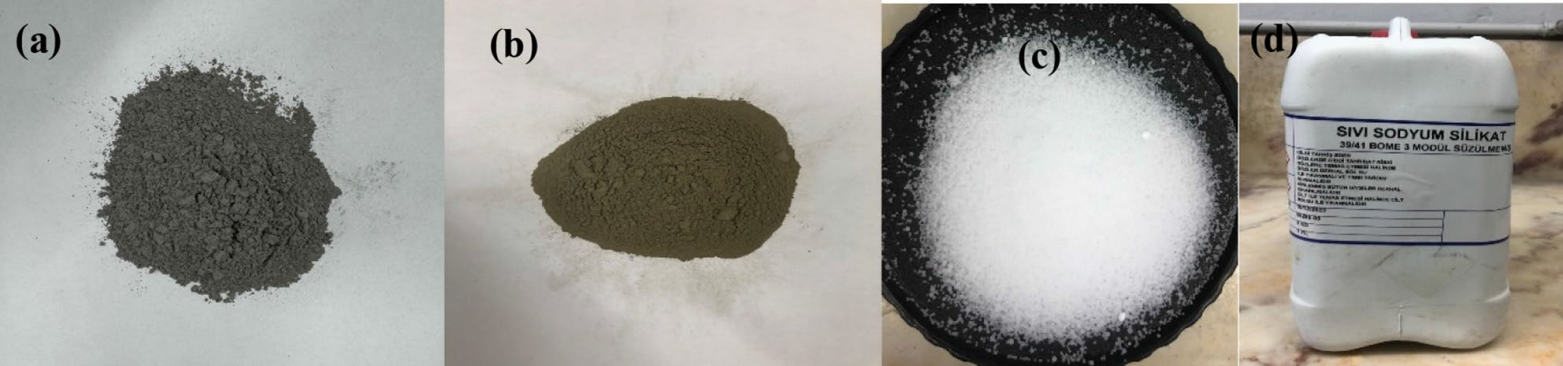
Raw materials used in produced specimens (**a**) FCS, (**b**) FA, (**c**) NaOH, (**d**) Na_2_SiO_3_).

Figure 1a–d shows waste FCS, FA and alkali activators (NaOH, Na_2_SiO_3_) used in the study. The results of the chemical analysis of the oxide compositions of FCS and FA were determined by X-Ray Fluorescence (XRF) spectroscopy using the Spectro Xepos II device operating at 50 kV and 0.7 mA accelerating voltage and are given in Table 1. The X-ray diffraction patterns of waste materials and the specimens were recorded on a diffractometer (Rigaku miniflex 600). The specimens were irradiated from Cu-Kα radiation at a voltage of 40 kV and current of 15 mA. The XRD analyses were carried out at a wavelength of 1.5406 (*λ*) between 10 and 90° at a step speed of 0.02° and a scanning speed at 2° per minute. The waste materials and the microstructural morphology of the produced specimens were analyzed using Hitachi SU3500 computer-controlled, 20 kV accelerating voltage digital SEM (scanning electron microscope of various scales).

**Table 1:** Chemical and physical composition of the waste materials according to XRF results.

Compounds	FCS	FA
SiO_2_	31.71	61.04
Al_2_O_3_	23.55	19.51
Fe_2_O_3_	2.31	8.76
CaO	0.60	2.96
MgO	33.86	1.65
SO_3_	0.37	0.21
Na_2_O	0.04	0.78
K_2_O	0.19	2.25
TiO_2_	0.35	–
P_2_O_5_	0.02	–
Cr	6.47	–
Mn_2_O_3_	0.15	–
Specific gravity	3.08	2.31
Specific surface area (m^2^/g)	0.362	1.01
LOI (%)	1.79	2.1

**Figure 2:**
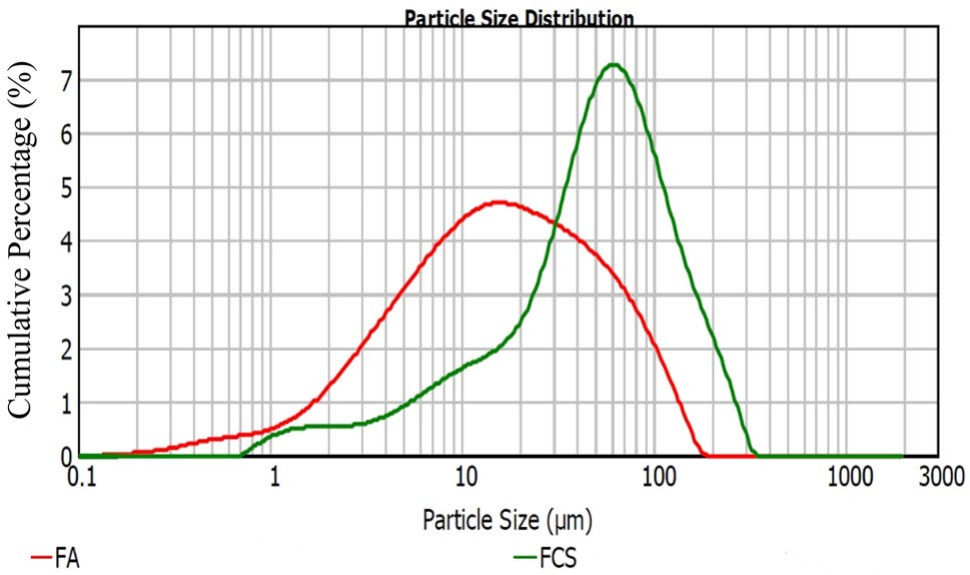
Particles sizes of the raw materials (FCS and FA).

**Figure 3:**
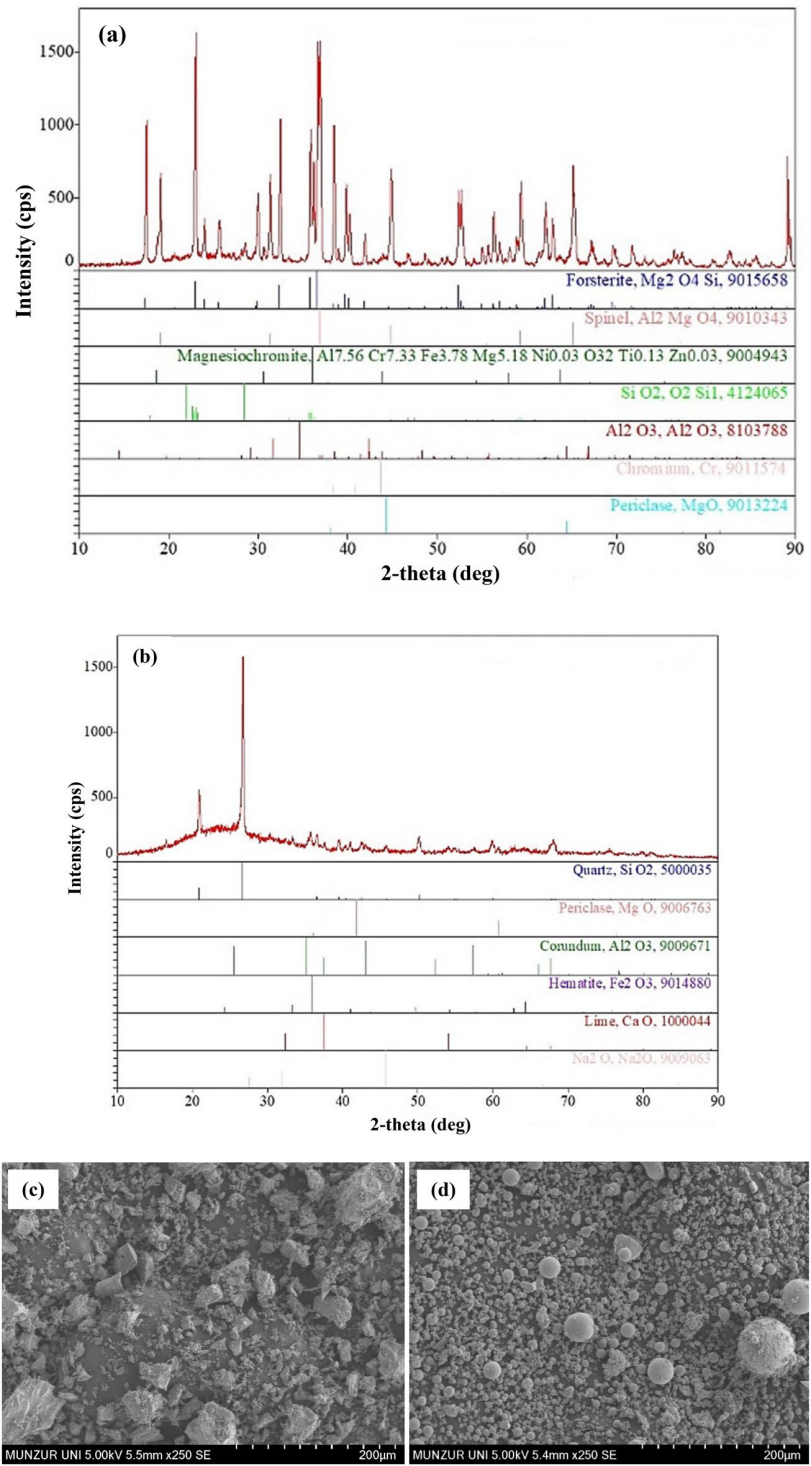
(**a**) XRD analysis of FCS, (**b**) XRD analysis of FA and (**c**) SEM images of FCS, (**d**) SEM images of FA.

Particle size distributions of waste FCS and FA raw materials are shown in Fig. 2 using the MALVERN/Mastersizer 2000 model device. Figure 3a–d show XRD analysis of the waste materials (FCS and FA) used in the specimen production and SEM images, respectively. The Blaine fineness value of the ground FCS was measured as 3730 cm^2^/g. As a result of the XRD experiment, the glassy phase contained in the slag was approximately 40%. In Fig. 3a, the crystalline phases in FCS are Forsterite (Mg_2_SiO_4_), Spinel (MgAl_2_O4), and Magnesiochromite ferroan ((Mg, Fe^2+^)(Cr,Al)_2_O_4_). Moreover, XRD analysis of fly ash (Fig. 3b) indicated that the major peak in fly ash was the quartz having a high potency of 2*θ* = 27°, which is strengthened by the XRF data where 60.74% of SiO_2_ was identified. Mullite was the second main peak at different ranges of 2*θ* (17, 32, 33, 42, 50, and 61°). SEM analysis of raw precursor materials shows distinct differences in their microstructures. Fly ash is generally characterized by its smooth and spherical shape. Waste FCS have irregular acicular particle shapes (Fig. 3c,d).

## Preparation of geopolymer mortar

**Figure 4:**
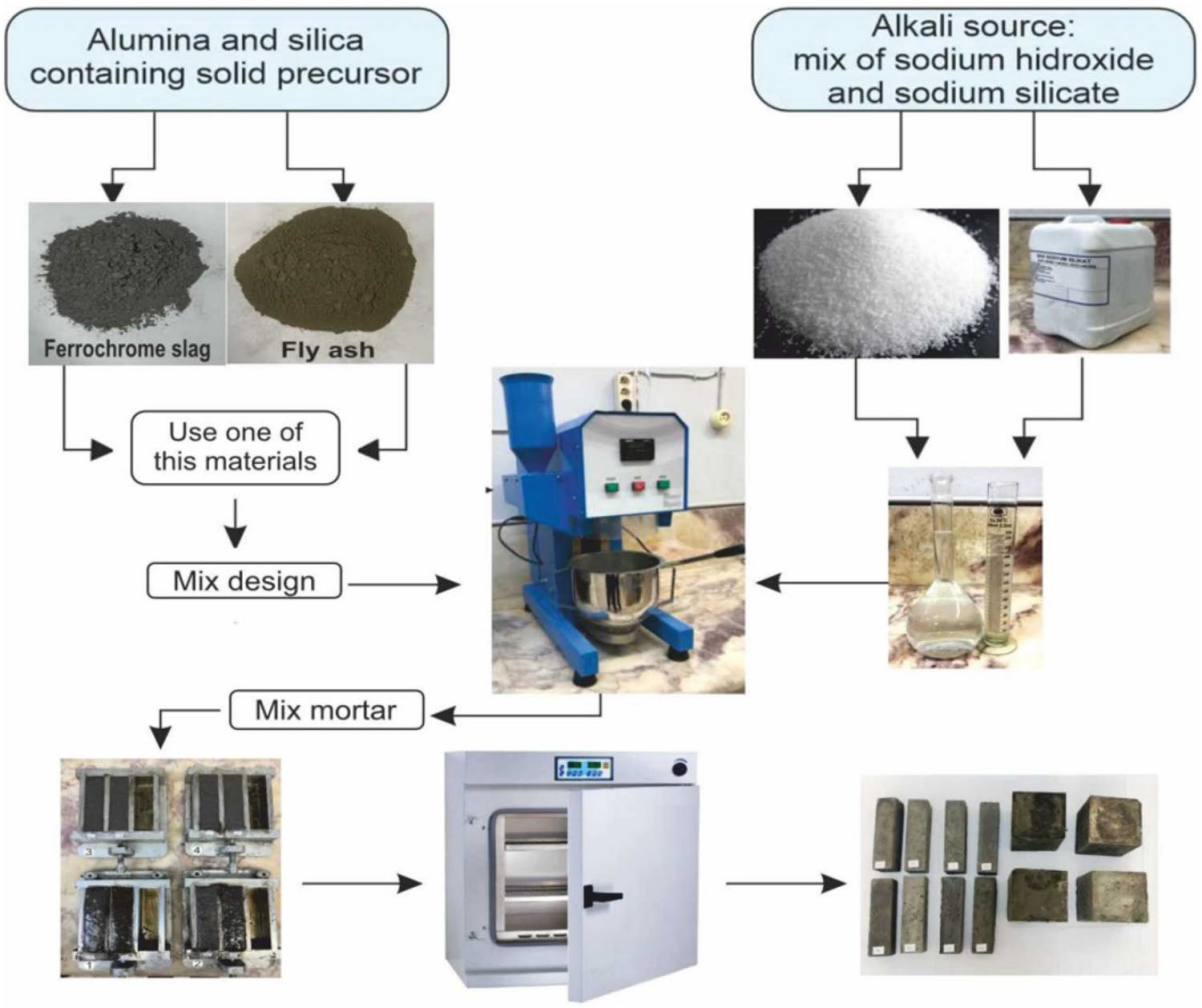
Flow chart of geopolymer mortar specimens production.

The methodology of the current study includes the production and laboratory experiments of an alternative building material that provides economic solutions and environmental and energy saving by mixing different ratios of metallurgical (ferrochrome slag) and thermal power plant wastes (fly ash) with alkaline activators (NaOH, Na_2_SiO_3_) instead of cement. Ferrochrome slag in the production of geopolymer mortar, was obtained from Eti Krom A.Ş. located in Kovancılar district of Elazığ province, was 3–5 cm in size and had irregular diameters and shapes. The slags brought from the plant were subjected to size reduction by passing through a jaw crusher before they were used in the study. Then, they were ground in a laboratory type ball mill and sieved in a sieve shaking device and FCS with a sub-sieve size of 200 µm was used as aggregate. According to TS EN 1097-6, the grain density is 3.08 gr/cm^3^^48^. The density of fly ash was measured as 2.31 gr/cm^3^ with the help of a pycnometer and was used as an additive to provide binding in the study. NaOH and Na_2_SiO_3_ were used together as activators and sintering aids to dissolve silica and alumina in FA and FCS powder, which are Al_2_O_3_-SiO_2_ sources, to form an aluminosilicate network in the mixture. NaOH was obtained from Zag Kimya Sanayi in Türkiye as granules and Na_2_SiO_3_ was obtained from Palkim Kimya Sanayi in Türkiye as 3 modules of liquid with a Baume range of 39°–41°. The choice of FCS and FA in the current study was necessitated by the increasing need for environmental sustainability through waste utilization, utilization of factory spaces and recycling of these waste materials for use in building materials. Although activators such as NaOH, KOH and K_2_SO_3_ have been used separately as sintering aids in a number of previous studies in building materials, the results related to FCS and FA with different Na_2_SiO_3_/NaOH ratios were investigated in this study. Before preparing the mixture for specimen production, the amounts of solution to be used as alkali activators (NaOH and Na_2_SiO_3_) were calculated. The choice of the ingredients of materials for geopolymer concrete helps develop better structural performances of geopolymer concrete^49^. In order to investigate the hybrid use of FA and FCS in alkaline activated mortars and to determine the optimum amount of FCS/FA, different mixing ratios were preferred in the study. In addition, the reason for choosing the FA used in the study in the range of 10–40% is to involve sufficient amounts of Si and Al to the reaction in the geopolymerization process and to see whether or not FA provides appropriate hydration products by showing pozzolanic effect and how this affects the structural performance. Some studies have reported that more than 40% FA negatively affects the structural performance of geopolymer concretes.^50,51^. The reason for choosing FCS in the range of 90–60% is that waste slag is more utilized and economical. The primary source of these materials composition consists of substantial reactive Si and Al oxide, leading to a better polymerization rate. Composite binder FA-FCS activators are reacted with NaOH and Na_2_SiO_3_ in the presence of SiO_2_ and Al_2_O_3_, dissolution of Si and Al ion releasing SiO_4_, and AlO_4_ into the solution during polymerization^52,53^. Since excessive use of NaOH and Na_2_SiO_3_ may reduce the mechanical strength of geopolymer mortars, NaOH solution was selected as 5 M and 10 M, which are widely used in the literature^54,55^. As the SS/SH ratio reduces, the structural performance of geopolymer concrete decreases due to sodium silicate reduction in the geopolymer reaction^56,57^. The solutions were prepared by mixing granular NaOH calculated according to the molarities to be used in the solution with 5 lt water. The solutions were kept at room temperature for about 24 h until they reached room temperature in order to reduce the effects of the solution heat caused by the exothermic reaction between NaOH and water. They were stirred in a magnetic stirrer for 5 min before they were used. Before using the Na_2_SiO_3_ solution, the Na_2_SiO_3_/NaOH ratio was determined as 1 and 2 and kept in separate glass bottles for use in the mixture. The specimen formulation contained FCS and FA at varying percentages by weight and mixtures of NaOH and Na_2_SiO_3_/NaOH as shown in Table 2. The aluminosilicate-based waste materials (FCS and FA) used in the mixture were mixed in different ratios in a mortar mixer at low speed for 2.5 min. Then, 5 M and 10 M NaOH solutions, which were brought to room temperature, were gradually poured into the mixer according to the desired mixing ratios and mixed at the same speed for another 2.5 min. Na_2_SiO_3_ solution was added to the homogenized mixture with Na_2_SiO_3_/NaOH ratio of 1 and 2 and mixing was continued at a lower speed for 2.5 min. Finally, the mixer was run at high speed for another 2.5 min in order for the mixture to have the desired homogeneity. The alkaline solution to binder (A/B) ratio of 0.35 and binder to fine aggregate ratio of 1:3 was considered in the present study. Figure 4 shows the flow chart showing the production stages of the geopolymer specimens.

**Table 2:** Mixture ratios of materials used in produced specimens.

Mixes ID	FCS (%)	FA (%)	NaOH	Na_2_SiO_3_/NaOH
A1	90	10	5 M	1
A2	80	20	5 M	1
A3	70	30	5 M	1
A4	60	40	5 M	1
B1	90	10	5 M	2
B2	80	20	5 M	2
B3	70	30	5 M	2
B4	60	40	5 M	2
C1	90	10	10 M	1
C2	80	20	10 M	1
C3	70	30	10 M	1
C4	60	40	10 M	1
D1	90	10	10 M	2
D2	80	20	10 M	2
D3	70	30	10 M	2
D4	60	40	10 M	2

**Figure 5:**
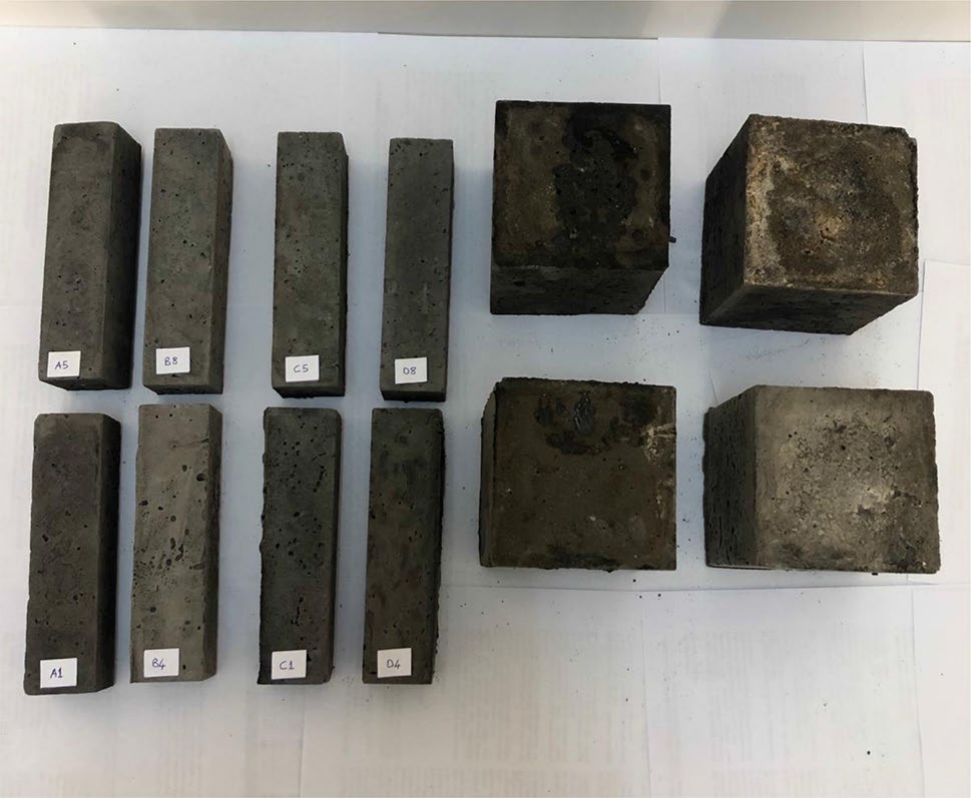
Specimens used in experimental.

The prepared mixtures were placed in 40 × 40 × 160mm prismatic molds for thermal tests and 100 × 100x100mm cube molds for mechanical tests according to TS EN 12,390-1^58^ by compacting with a tamping rod. Figure 5 shows the produced geopolymer mortar specimens. The geopolymer mortars were cured in an oven at 70 °C and 100 °C for 24 h. After 24 h, the geopolymer mortars were removed from the molds and air cured under ambient conditions (20 ± 2 °C) until the experiment day (28 days). The specimens were then subjected to thermal and mechanical tests.

### Testing procedure

#### Bulk density and apparent porosity testing

Bulk density and apparent porosity values of 40 × 40 × 160 mm specimens were measured using Archimedes’ principle depending on ASTM C20-00 standard. After the dried weight of the specimens was measured, they were soaked in boiling water for three hours. Their soaked weights were then measured. They were suspended in water through a spring balance. Their last weights were measured, as well.

The following formula was used to calculate the bulk density:

1 $${\rho b} = {\text{W}}_{1} \times {\rho w}/{\text{W}}_{2} - {\text{W}}_{3}$$

The following formula was used to calculate the apparent porosity:

2 $${\text{A}} = {\text{W}}_{2} - {\text{W}}_{1} /{\text{W}}_{2} - {\text{W}}_{3}$$

where A is apparent porosity, ρb is bulk density, W_1_ is dry weight of the specimen, ρw is density of the water (g/cm^3^), W_2_ is soaked weight of the specimen in water, and W_3_ is Suspended weight of the specimen in water.

#### Water absorption testing

Water absorption values of 40 × 40 × 160 mm specimens were measured using Archimedes’ principle depending on ASTM C20-00 standard. The specimens were boiled in water at 100 °C for two hours and soaked in water for additional four hours in order to run the water absorption test. The following formula was used to calculate the water absorption:

3 $${\text{W}}_{{\text{a}}} = {\text{ W}}_{{\text{s}}} - {\text{W}}_{{\text{d}}} /{\text{ W}}_{{\text{d}}} \times \, 100$$

where W_a_ is percentage absorption of water by the specimen, W_s_ is soaked weight of the specimen after boiling at 100 °C for two hours, and W_d_ is dry weight of the specimen.

#### Compressive strength testing

The compressive strength tests were run on the specimen in accordance with the TS EN 772-1 standard, using a BZ 701/120 compressive strength device (is equipped with a digital command panel and an adjustable loading rate and can apply force at a single axis). Cubic specimens having a size of 100 × 100 × 100 mm were utilized and their compressive strength was read digitally.

#### Thermal conductivity testing

The thermal conductivity test was run on 40 × 40 × 160 mm specimens. Their thermal conductivities, specific heats and thermal diffusivity were assessed by using a Isomet 2104 portable heat transfer analyzer. This device measures based on the hot wire method in accordance with Norm (DIN) 51,046. Measurements were made on various parts of the specimens three times and the average values of these measurements were taken. Its range was 0.02–6.00 W/mK and its sensitivity was ± 5% precision and volumetric heat capacity was in the range of 4.0 × 10^4^ J/m^3^ K and 4.0 × 10^6^ J/m^3^ K with 15% precision. The temperature ranged between 26 and 28 °C.

## Result and discussion

### Bulk density and apparent porosity

**Figure 6:**
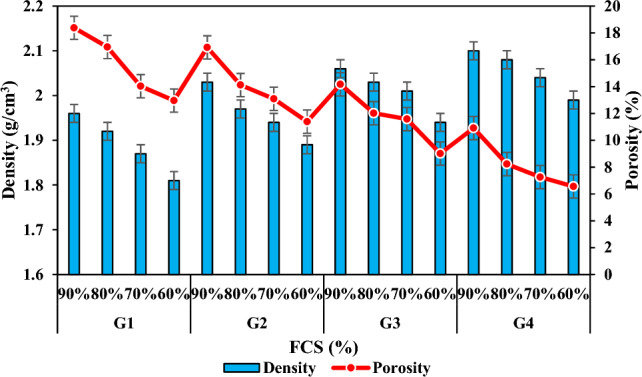
Bulk density and apparent porosity variation according to the waste material ratio in the specimens.

The proper physical properties of the product to be produced vary depending on the composition of the solid raw material, the reaction of the relevant chemicals by mixing them in the required molar ratios, the Si/Al ratio, the heat and duration of the thermal curing or calcination process applied.^59^. Density and porosity were found to be the two most important parameters that can control many physical properties of geopolymer mortar specimens produced in the present study. The porosity refers to the total volume percentage of the pores in the specimen. When the results (Table 3 and Fig. 6) related to the density and porosity of the specimens were analyzed, it was observed that they were in a decreasing change according to the decrease of FCS ratio and the increase of FA ratios. The densities of the specimens in the groups G1, G2, G3 and G4 decreased by 7.65%, 6.89%, 5.82%, and 5.23% and their porosities decreased by 29.48%, 32.68%, 36.41%, and 39.87% respectively. The first reason for this is that the alkaline silica gel formed by the reaction of alkaline active solution and FA particles reacted with FCS and formed a structural bond, and the second reason can be attributed to the decrease in the difference between their specific gravities in the FA addition by decreasing FCS with high density as well as the pore structure formed^60–63^.

**Table 3:** Experimental results of the specimens.

Group	Specimens Code	NaOH (M)	Na_2_SiO_3_/NaOH (SS/SH)	Bulk Density (g/cm^3^)	Porosity (%)	Thermal Conductivity (W/mK)	Water Absorption (%)	Compressive Strength (MPa)
			Oven dried 70 °C					
	A1	5	1	1.96	18.38	0.428	11.16	8.23
G1	A2	5	1	1.92	16.95	0.491	10.35	10.14
	A3	5	1	1.87	14.03	0.512	9.81	14.22
	A4	5	1	1.81	12.96	0.586	8,81	19.85
	B1	5	2	2.03	16.92	0.484	9.08	9.03
G2	B2	5	2	1.97	14.12	0.521	8.58	13.25
	B3	5	2	1.94	13.09	0.549	8.41	16.39
	B4	5	2	1.89	11.39	0.607	6.85	17.52
	C1	10	1	2.06	14.17	0.553	7.95	11.12
G3	C2	10	1	2.03	12.02	0.592	6.91	15.55
	C3	10	1	2.01	11.58	0.613	5.83	17.13
	C4	10	1	1.94	9.01	0.674	4.86	20.98
	D1	10	2	2.10	10.91	0.588	6.02	13.02
G4	D2	10	2	2.08	8.23	0.627	4.31	16.42
	D3	10	2	2.04	7.26	0.686	3.92	17.31
	D4	10	2	1.99	6.56	0.691	3.48	18.78
			Oven dried 100 °C					
	A5	5	1	1.84	20.15	0.398	12.27	10.45
G5	A6	5	1	1.81	18.18	0.412	11.96	12.39
	A7	5	1	1.77	17.32	0.488	11.42	16.41
	A8	5	1	1.72	15.13	0.527	10.42	21.13
	B5	5	2	1.91	18.66	0.411	10.71	12.12
G6	B6	5	2	1.87	17.41	0.492	10.19	15.85
	B7	5	2	1.83	14.28	0.501	10.02	18.27
	B8	5	2	1.79	12.97	0.549	9.16	19.98
	C5	10	1	1.97	15.48	0.462	9.92	15.02
G7	C6	10	1	1.94	14.95	0.505	8.94	18.89
	C7	10	1	1.89	13.54	0.579	8.52	19.69
	C8	10	1	1.86	10.88	0.602	7.26	23.92
	D5	10	2	2.01	12.78	0.511	8.38	17.73
G8	D6	10	2	1.98	11.25	0.549	6.12	20.76
	D7	10	2	1.94	9.41	0.617	5.88	21.07
	D8	10	2	1.92	8.81	0.639	5.42	21.95

**Figure 7:**
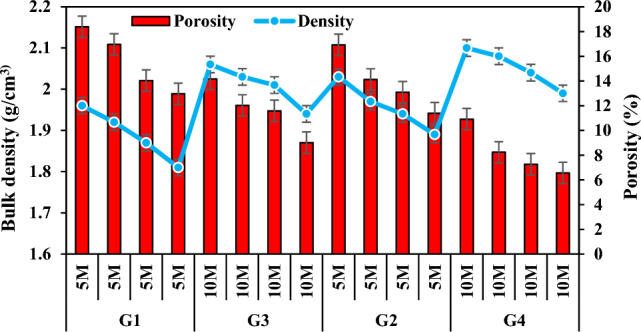
Bulk density and apparent porosity variation according to molarity increase.

When the experimental results and Fig. 7 were analyzed, it was observed that the bulk density values of the mortar specimens prepared at 5 M NaOH concentration were lower than the bulk density values of the mortar specimens prepared at 10 M concentration and higher than the porosity values. In other words, as the molarity increased, bulk density values increased and porosity values decreased. With the increase in molarity, the densities of the specimens in the group G3 increased by 5.72–7.48% compared to the densities of the specimens in the group G1, while their porosities decreased by 17.46–30.47%. While the densities of the specimens in the group G4 increased by 3.44–5.58% compared to the densities of the specimens in the group G2, their porosities decreased between 35.52 and 44.53%. The reason for this is that when the molarity increases, the dissolution of the existing silicate and aluminate minerals increases and thus the shape and number of unreacted fly ash decreases, the bond strength between the molecules increases, the homogeneous structure increases and the hollow structure is removed^64^.

**Figure 8:**
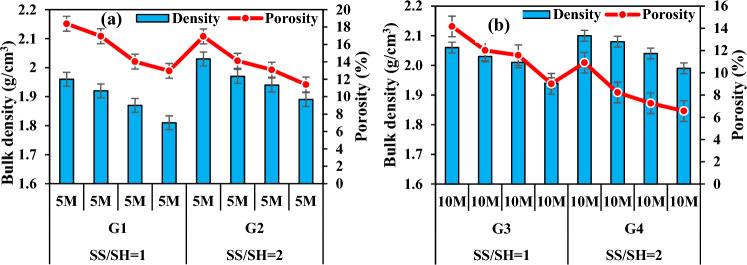
Bulk density and apparent porosity variation according to SS/SH ratio.

When the correlation between bulk density and porosity of the specimens with the same molarity and SS/SH was analyzed (Fig. 8a,b), it was observed that bulk density increased and porosity decreased when SS/SH ratio increased. When the SS/SH ratios were increased from 1 to 2, the density values of the specimens in the groups G1 and G2 increased by approximately 2.53–4.23% while their porosities decreased by 6.69% and 16.70%. The density values of the specimen in the groups G3 and G4 increased approximately between 1.47 and 2.51% while their porosities decreased by 23.01–37.30%. This can be caused by the fact that the SS activator has a higher density value than the SH solution. Also, increasing the percentage of Na_2_SiO_3_ results in higher SiO_2_ than Al_2_O_3_ and thus more Si–O–Si bonds, which are considered to be stronger than Si–O–Al bonds^65^. This results in a denser structure with less pores. Interestingly, in their recent study, Ma et al.^66^ determined that the addition of Na_2_SiO_3_ to a solution of alkali could increase the Si to Al ratio, leading to a low porosity and a finer pore network in a geopolymer matrix.

**Figure 9:**
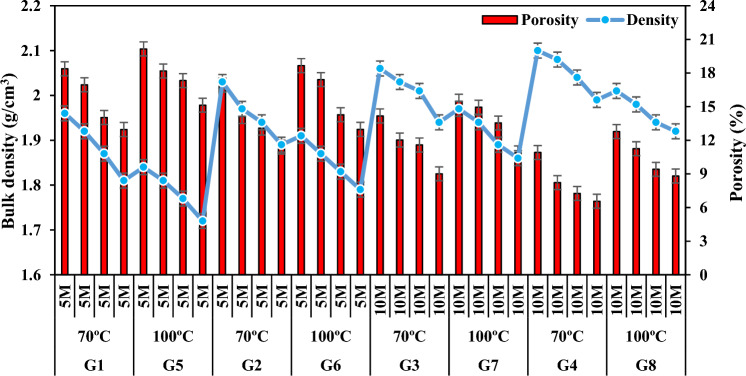
Bulk density and apparent porosity variation of specimens according to firing temperature.

According to the results obtained from the studies conducted by the researchers, pores and voids in geopolymer mortars can generally be caused by the presence of air bubbles during the mixing and casting process and the evaporation of water during the curing process. In addition, cracks may also occur due to shrinkage caused by curing at high temperature. Thus, the optimum temperature values of 70 °C and 100 °C were selected as a result of the experiments. Figure 9 shows that when the curing temperature was increased from 70° to 100 °C, the bulk density values of the specimens decreased while the porosity values increased. When the density values of the groups G1-G5, G2-G6, G3-G7 and G4-G8 were compared according to the increase in curing temperature, it was observed that the density values decreased by 12.24%, 11.82%, 9.70%, and 8.57%, respectively, while the porosity values increased by 35.68%, 38.96%, 41.79%, and 48.66%, respectively. The first reason for this is the evaporation of unreacted water in the specimen during geopolymerization due to the increase in curing temperature. The second reason is that the increase in curing temperature weakens the amorphous structure of the geopolymer and the Si–O–Al bonds of fly ash particles that provide alumina-silicate source to the matrix. Thus, the fly ash expands and disintegrates in the matrix and decomposes and makes the specimen lighter and more voided over time.

### Water absorption

**Figure 10:**
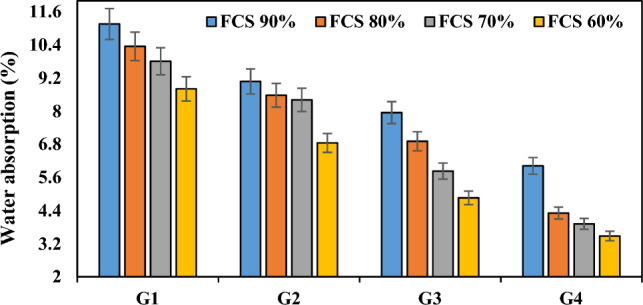
Variation of water absorption capacity according to waste material ratio.

Performance of geopolymer composites were dependent on the porosity and water absorption percentage^67^. When the pores are disconnected and smaller, the percentage of water absorption is respectively low. Water absorption is the primary mechanism for water and water vapor transport in concrete The lower the water absorption characteristics the concrete has, the longer its lifespan. According to the experimental results in Table 3, it was found out that the water absorption values of the mixtures depended on the varying ratios of waste FCS and FA, and also NaOH molarity, SS/SH ratios and curing temperatures played an effective role in water absorption values. Figure 10 shows the variation of the water absorption test results according to the waste FCS and FA ratio. When Fig. 10 was analyzed, it was concluded that the water absorption ratios decreased with the decrease in FCS utilization ratio, i.e. with the increase in fly ash use ratio. The water absorption values of the specimens in the groups G1, G2, G3, and G4 decreased by 21.05%, 24.55%, 38.86%, and 42.19%, respectively. The first reason for this can be attributed to the reduction of the amount of FCS^68^ in the specimen, which has a high water absorption ratio (13.63%) due to its mineral structure, and the second reason can be attributed to the low CaO content in the FA and the interlocking of the crystals formed by a developed interfacial transition zone that provides a good adhesion between FCS and mortar matrix in the microstructure.

**Figure 11:**
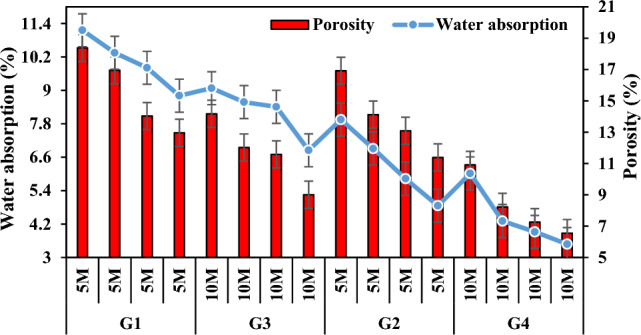
Variation in water absorption capacity according to molarity increase and its relationship with apparent porosity.

Given that geopolymers are more resistant to outdoor conditions and the chemicals in the geopolymer are less likely to rise to the geopolymer surface, it is very important that the water absorption value is low. Table 3 lists and Fig. 11 shows the changes in water absorption values by increasing the NaOH concentration in the specimens from 5 to 10 M and they were found to be similar to the changes obtained from porosity experiments. It was observed that the water absorption ratios decreased with the increase in molarity value in the specimens. With the increase in molarity, the water absorption ratios of the specimens in the group G3 decreased by 28.76–44.83% compared to the water absorption ratios of the specimen in the group G1, and the specimens in the group G4 decreased by 33.70–53.38% compared to the specimens in the group G2. This is primarily associated with the low strength and porous morphology of the fly ash geopolymer which reduces the porosity at 10 M^69^. Moreover, the water absorption of GPC reduces with increasing molarity of NaOH because of reduced water contents^70,71^. The fly ash particles mixed with viscous alkaline activators lead to viscous and cohesive GPC^72^. The water content has an important role in the dissolution of ions in geopolymerization process^73^. As NaOH concentration increased, the increase in Si^+^ and Al^+^ ions dissolved in the sample triggered more geopolymer formation by providing better reactivity with fly ash. Some studies also noted a decrease in the water absorption in the specimen with increasing NaOH concentration^74,75^. They revealed lower water absorption and apparent porosity when higher alkali content (% Na_2_O) activators were used to make geopolymer mortars^69,76^.

**Figure 12:**
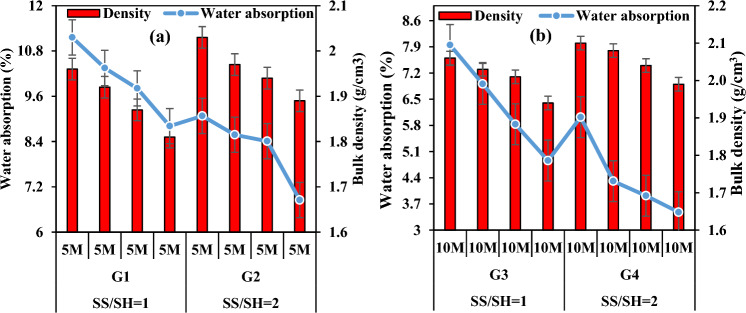
Water absorption capacity variation according to SS/SH ratio and its relationship with bulk density.

When the amount of water in activator solutions and the amount of activator required for a certain consistency are considered, it is understood that the amount of water is an important factor affecting the fluidity of geopolymeric mixtures. The amount of water and the humidity of the curing environment also affect strength development. Figure 12a,b shows the correlation between the water absorption and bulk density ratios of the specimens and the SS/SH ratio. Geopolymeric mortar specimens made using a ratio of Na_2_SiO_3_/NaOH up to 2 appeared to cause lower water absorption ratios and porosity compared to those up to 1. From Fig. 12, it was observed that increasing the SS/SH ratio in the specimens with unchanged FCS and FA addition ratios increased the density and caused a further reduction in the water absorption ratios. When the SS/SH ratio was increased from 1 to 2, the water absorption ratios of the specimens in the groups G1 and G2 decreased by 14.27–22.25%, and the water absorption values of the specimens in the groups G3 and G4 decreased by about 24.28–37.63%. This finding is compatible with those reported by the researchers finding that high dissolution of the FA particles in the mixture via higher alkali content resulted in denser microstructure in geopolymers^69^.

**Figure 13:**
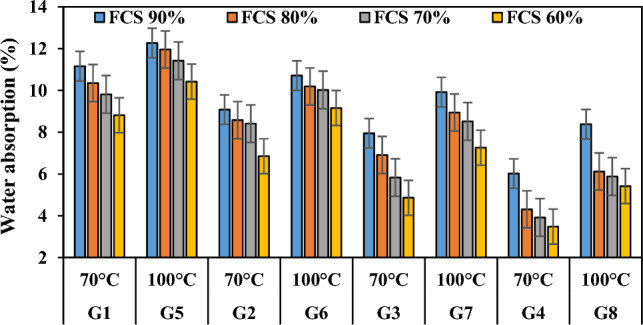
Water absorption capacity variation of specimens according to firing temperature.

Curing temperature and method are very important parameters that determine the water absorption efficiency in the geopolymerization reaction^77^. Figure 13 shows that hot curing conditions slightly increased the water absorption values. When the water absorption ratios of the groups G1-G5, G2-G6, G3-G7 and G4-G8 were compared, it was found that the water absorption ratios increased by 9.95–18.27%, 17.95–33.72%, 24.78–49.38% and 39.20–55.75%, respectively. This indicates that the geopolymerization process between the binder and the alkaline activator is a thermal phenomenon and it is necessary to determine the optimum temperatures for the determination of the dense geopolymer matrix with the formation of the polymer chain of aluminosilicate bonds. The specimens with the highest water absorption value were those cured at 100 °C. This was associated with the rapid reaction of the specimens when cured at high temperatures and the rapid evaporation of water in the structure and the excess of gaps opened by the evaporation path. Thus, in the present study, it can be asserted that the degree of geopolymerization that allows the formation of a less dense and porous matrix is reached when the temperature is increased from 70° to 100 °C.

The critical moisture content is 30% of the total dry volume and the material does not deform below this ratio during freezing^78^. The highest water absorption ratio was obtained from the specimen A5 produced at 100^0^C and the lowest water absorption ratio was obtained from the specimen D4. The water absorption ratios of the specimens were lower than the critical value (30%). Thus, low water absorption reduces the cracking and shrinkage potential of concrete. Thus, the concrete obtained can be used in areas directly exposed to water. If FCS, FA or AA are used at higher ratios than the ratios in the current study when producing FCS and FA-doped geopolymer mortars, water absorption ratios will increase even more. In this case, disintegration will occur in the produced geopolymers due to micro cracks. Thus, the ratios used in the present study are sufficient to produce geopolymer mortars with high permeability. As a result, as geopolymer structures do not directly hold water, they can partially prevent water losses in concrete from damaging cement structures.

### Compressive strength

**Figure 14:**
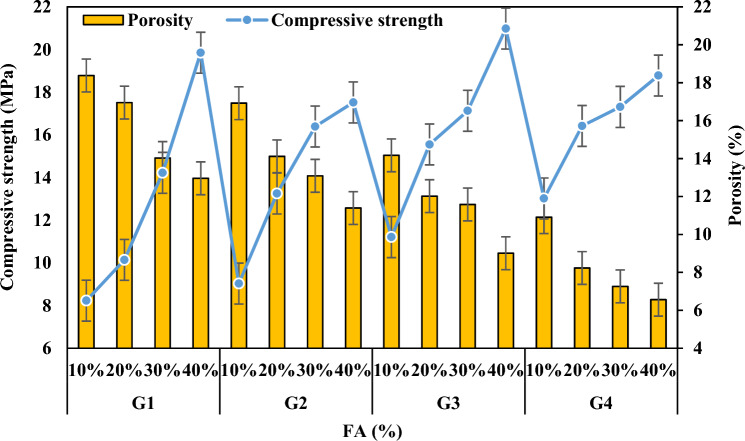
Variation in compressive strength according to waste material ratio and its relationship with apparent porosity.

The enhancement in compressive strength observed in the produced geopolymer mortars can be attributed to the intricate bonding mechanism between the waste FCS and the geopolymer matrix, facilitated by the FA binder. This bonding mechanism is governed by several key parameters, including the alkali liquid/binder ratio, the type and concentration of the alkali solution, and the molar ratio of SiO_2_ to Na_2_O. While the precise impact of each parameter on GPC remains incompletely understood, certain well-established trends have been identified^79,80^. It is established that strength diminishes with an increase in the alkali liquid/binder ratio and conversely improves with higher concentrations of alkaline activators. Additionally, an optimal molar ratio of SiO_2_ to Na_2_O, typically ranging between 1.0 and 2.0, is deemed favorable for achieving optimal compressive strength. In the current study, a reduction in the waste FCS ratio, consequently elevating the FA ratio, led to a notable decline in porosity alongside a concurrent increase in compressive strength (as illustrated in Fig. 14). The compressive strengths of specimens across different FCS and FA ratios exhibited considerable enhancements. Specifically, specimens in groups G1, G2, G3, and G4 demonstrated increases of 58.54%, 48.46%, 46.57%, and 30.67%, respectively. Similarly, specimens in groups G5, G6, G7, and G8 exhibited improvements of 50.54%, 39.33%, 37.20%, and 19.22%, respectively. This augmentation can be primarily linked to a reduction in the release rate of silica and alumina ions owing to the sluggish dissolution kinetics of FCS. Additionally, the presence of starting materials with a low glassy percentage in the crystal structure, such as forsterite and spinel, may impede the reactivity and diminish the potential of the geopolymer reaction.

**Figure 15:**
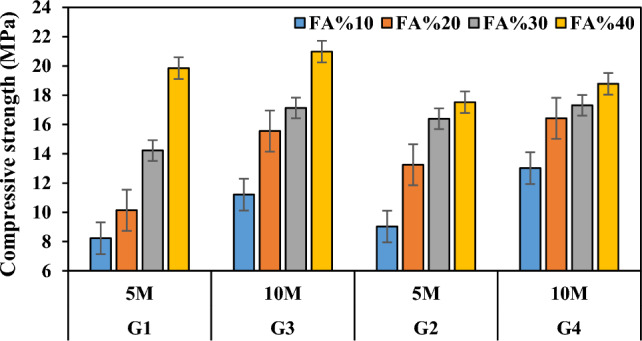
Variation of compressive strength according to waste material ratio and molarity increase.

FCS is different from typical geopolymer precursor materials as it contains more than 30% MgO. In the present study, when the slag was tested by mixing with alkaline containing materials and chemicals, it was found that it reached compressive strengths comparable to Portland cement mortars. Table 3 lists and Fig. 15 shows the compressive strengths of the specimens produced according to the NaOH concentration ratio. With the increase in molarity, the compressive strengths of the specimens in the group G3 increased by 5.69%-53.35% compared to the compressive strengths of the specimens in the group G1, and the compressive strengths of the specimens in the group G4 increased by 5.61–44.18% compared to the specimens in the group G2. The compressive strength values of the specimens with 5 M concentration ranged between 8.23 and 19.85 MPa, while the compressive strength values of the specimens with 10 M concentration increased and ranged between 11.21 and 20.98 MPa. The compressive strength values increased according to the activator ratio in the mixture. The positive effect of increasing SH molarity on the strength development of lightweight geopolymer mortars can be explained by the dissolution of a larger amount of fly ash particles. Similar results were obtained by the authors in studies focusing on geopolymers^81–85^. When NaOH solution more concentrated than 5 M was used, the strengths obtained increased slightly. High alkali concentration is generally associated with improved compressive strength^86,87^. However, excessive molarity can reduce ion mobility, which slows down the geopolymer reaction^88^. Additionally, alkali ratio controls the amount of liquid in the mixture, thus lower values produce stronger concrete because of the relative increase in binder content^89,90^. Naturally, as NaOH increases, more bond formation with silicates and aluminates can be observed. This is thought to be due to the reaction of silicates and aluminates to isolate the voids. However, in order for the alkaline activator to form bonds when it exceeds a certain ratio, especially the aluminum oxide ratio in the raw material must be high.

**Figure 16:**
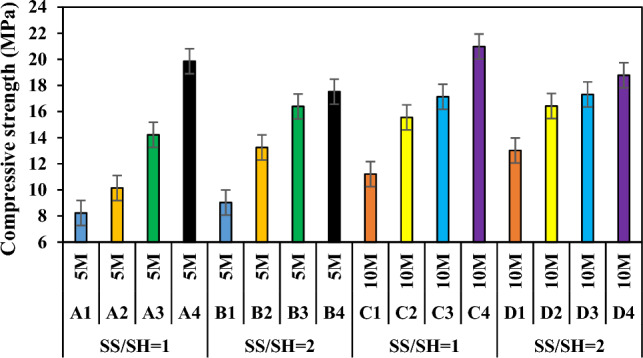
Compressive strength variation according to SS/SH ratio of specimens with the same molarity.

The ratio of sodium silicate to sodium hydroxide (SS/SH) plays a critical role, as both low and high levels of Na + can affect compressive strength. Low Na + and OH^-^ inhibits complete dissolution and polymerization, and high Na + content weakens the geopolymer structure by leaving excess Na + residue in the specimen^91^. Figure 16 shows the correlation between SS/SH and compressive strength. The compressive strengths of each of the specimens with 5 M concentration increased when the SS/SH ratios were increased from 1 to 2, but decreased from 19.85 to 17.52 MPa when the specimens A4 and B4 were compared. When the SS/SH ratios of each of the specimens with 10 M concentration were increased from 1 to 2, the compressive strengths increased but decreased from 20.98 to 18.78 MPa when the specimens C4 and D4 were compared. The compressive strength of geopolymer mortar mixtures is more likely to increase with SS/SH until a certain maximal point and drops. This could be because increasing Si–O bonds significantly increased the strength of the geopolymer mortar; whereas excess, unreacted silica inhibited water evaporation and structure formation, resulting in a reduced strength in the geopolymer^89,92,93^. It should be stated that very concentrated basic solutions also have chemical caustic properties and reduce compressive strength.

**Figure 17:**
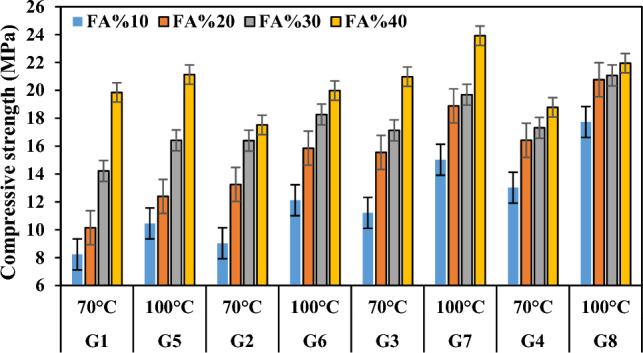
Compressive strength variation according to firing temperature.

Higher or lower temperature curing negatively affects the development of mechanical properties. Most of the studies have revealed that heat in the curing of geopolymer mortars and concretes helps to accelerate the geopolymer reaction to achieve compressive strength in the early stage, however, thermal curing has no significant effect in the later stages^94^. It is also observed that specimens produced with increasing sodium silicate ratio are more effective compared to sodium hydroxide, especially when high-temperature curing is applied. The compressive strengths increased when the curing temperature applied to the specimens increased from 70 to 100 °C (Fig. 17). When the compressive strengths of the groups G1-G5, G2-G6, G3-G7 and G4-G8 were compared, it was found that the compressive strengths increased by 6.44–26.97%, 11.47–34.21%, 14.01–33.98%, and 16.87–36.17%, respectively. In the current study, the compressive strength values of the specimens A4, B4, C3, C4, D2, D3, and D4 produced at 70 °C and the specimens A8, B7, B8, C6, C7, C8, D5, D6, D7, and D8 produced at 100 °C were higher than the minimum compressive strength value of 17.2 MPa recommended for building materials in ACI 318. Thus, these specimens can be preferred where they are intended to be used as carriers in buildings. The decrease in compressive strength of the other specimens with increasing curing temperature is related to shrinkage and micro-scale cracking due to dehydration and shrinkage of geopolymeric gel^95^. The highest compressive strength (23.92 MPa) was obtained from the specimen C8 at 100 °C and the lowest compressive strength (8.23 MPa) was obtained from the specimen A1 at 70 °C. Similar to the findings in the current study, it was reported that due to the increase in the geopolymerization reaction at 40 °C curing temperature, the unreacted fly ash started to disintegrate and N-A-S-H crystals were produced by increasing the curing temperature to 60 °C and as a result, high strength was obtained^96^.

### Thermal conductivity

**Figure 18:**
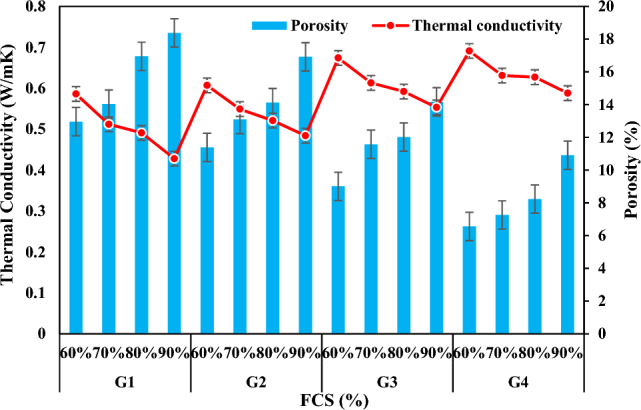
Thermal conductivity variation according to waste material ratio and its relationship with apparent porosity.

The buildings consume energy based on the thermal conductivity values of the building materials^97^. Thermal conductivity is the most important thermal property that has an effect on heat transfer by conduction through concrete^98^. Density and apparent porosity are related with thermal conductivity. Some studies have confirmed that there is an opposite relationship between density and thermal conductivity^99^. Moreover, as porosity increased, thermal conductivity decreased. This is because higher porosity means more voids and the thermal conductivity of air within the voids is much lower compared to solid substance, thus resulting in a relatively lower thermal conductivity in the whole material^100^. Furthermore, type, amount, and pore content of aggregates were factors affecting the mechanical and thermal properties of geopolymer concrete^101^. Table 3 lists the thermal conductivity values of the produced specimens and Fig. 18 shows the change of thermal conductivity with porosity. When waste FCS was increased and FA powder was decreased, porosity increased and thermal conductivity values decreased. Thermal conductivity values of the specimens in groups G1, G2, G3, and G4 decreased by 26.96%, 20.26%, 17.95%, and 14.90%, respectively. Among the specimens G1, G2, G3 and G4, the lowest thermal conductivity coefficient (0.428W/mK) was detected in the specimen A1 and the highest thermal conductivity coefficient (0.691W/mK) was detected in the specimen D4. This is associated with the fact that as FCS ratio increases, binder FA does not show exact adhesion with FCS and therefore porosity in the geopolymer matrix increases.

**Figure 19:**
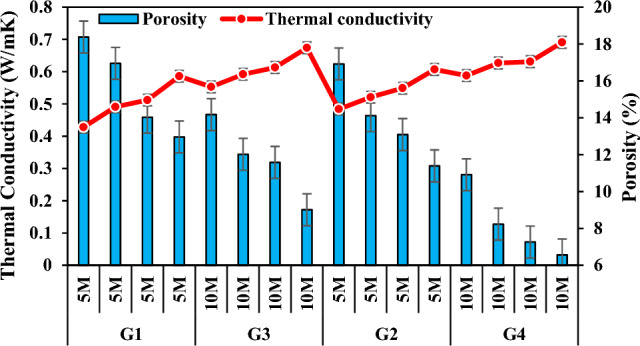
Thermal conductivity variation according to molarity increase and its relationship with apparent porosity.

Figure 19 shows the change of thermal conductivities of the specimens according to NaOH concentrations and porosity ratios. As the molarity increased, the density increased and the porosity decreased, thus the thermal conductivities increased. With the increase of molarity, the thermal conductivity values of the specimens in the group G3 increased by 15.01–29.20% compared to the thermal conductivity values of the specimens in the group G1, and those of the specimens in the group G4 increased by 13.83–21.48% compared to the specimens in the group G2. The thermal conductivity values of the specimens with 5 M concentration increased by 4.28W/mK–0.607W/m and the values of those with 10 M concentration increased by 0.553W/mK–0.691W/mK. The reason for this may be that NaOH has a higher density and a denser and less porous structure forms by increasing the concentration^102^. Thus, specimen with better thermal insulation can be produced with low NaOH molarity.

**Figure 20:**
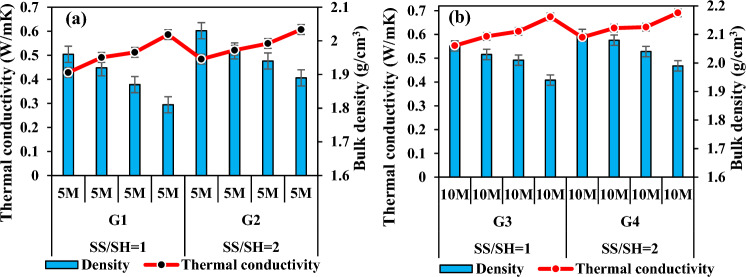
Thermal conductivity variation according to SS/SH ratio and its relationship with bulk density.

Figure 20a,b presents a detailed analysis of the thermal conductivity values of specimens activated with 5 M and 10 M concentrations, delineated against the density values. Upon augmenting the Na_2_SiO_3_ content, the thermal conductivity values exhibited discernible fluctuations. Notably, as the SS/SH ratio transitioned from 1 to 2, a noteworthy increase in thermal conductivity was observed across the spectrum of specimens. Specifically, thermal conductivity values in groups G1 and G2 demonstrated an augmentation ranging from approximately 3.46 to 11.57%, while those in groups G3 and G4 displayed a more modest increase, ranging from about 2.46 to 5.95%. Interestingly, specimens produced by elevating the Na2SiO3 content within the same molarity exhibited a comparatively lower rise in thermal conductivity values compared to those generated through an increase in molarities. This discrepancy can be attributed to the inherent density differential between Na_2_SiO_3_ and NaOH, with the former possessing a lower density than the latter. Consequently, the interplay of varying densities between these alkali activators likely contributed to the observed differences in thermal conductivity values. Moreover, the most pronounced changes in thermal conductivity were evident in mixtures featuring a Na_2_SiO_3_ to NaOH ratio of 2, particularly when the NaOH concentration was augmented from 5 to 10 M. This finding underscores the significant impact of altering alkali activator ratios on thermal conductivity characteristics, particularly when considering the intricate interplay between different components within the geopolymer matrix.

**Figure 21:**
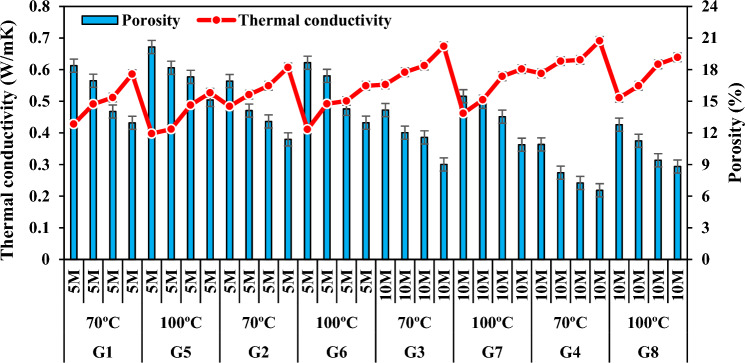
Thermal conductivity variation of specimens according to firing temperature and its relationship with apparent porosity.

Figure 21 shows the variation of thermal conductivity coefficients with porosity by increasing the curing temperature for all mixtures. When the thermal conductivity values of the groups G1-G5, G2-G6, G3-G7 and G4-G8 were compared, it was observed that they increased by 4.69–6.09%, 5.57–15.08%, 5.55–16.46%, and 2.22–13.10%, respectively. When the curing temperature was increased from 70 to 100 °C, the thermal conductivity of the specimens decreased more. The first reason for this can be the evaporation of the water in the specimen with the increase in temperature; the second reason can be the formation of Al–Si–H gel matrix with the increase in temperature, but the Al–Si–H gels cannot make a great contribution to the condensation with the increase in temperature and the porosity increases; the third reason can be explained by the interlocking of the needle-like crystals formed due to CaO and SiO_2_ in the materials used, which increases the strength in the structure, but the pores and voids remaining between them reduce the thermal conductivity coefficient. The lowest thermal conductivity value among all the specimens (0.398W/mK) was obtained from the specimen A5. The present study revealed that the specimens produced at low molarity and max. 100 °C curing temperature were more efficient in terms of carbon emission and thermal insulation due to their low thermal conductivity. In addition, the best concrete in terms of energy consumption was found in SS/SH:1 type.

### Microstructure examination of geopolymer mortar

#### SEM–EDS analysis

**Figure 22:**
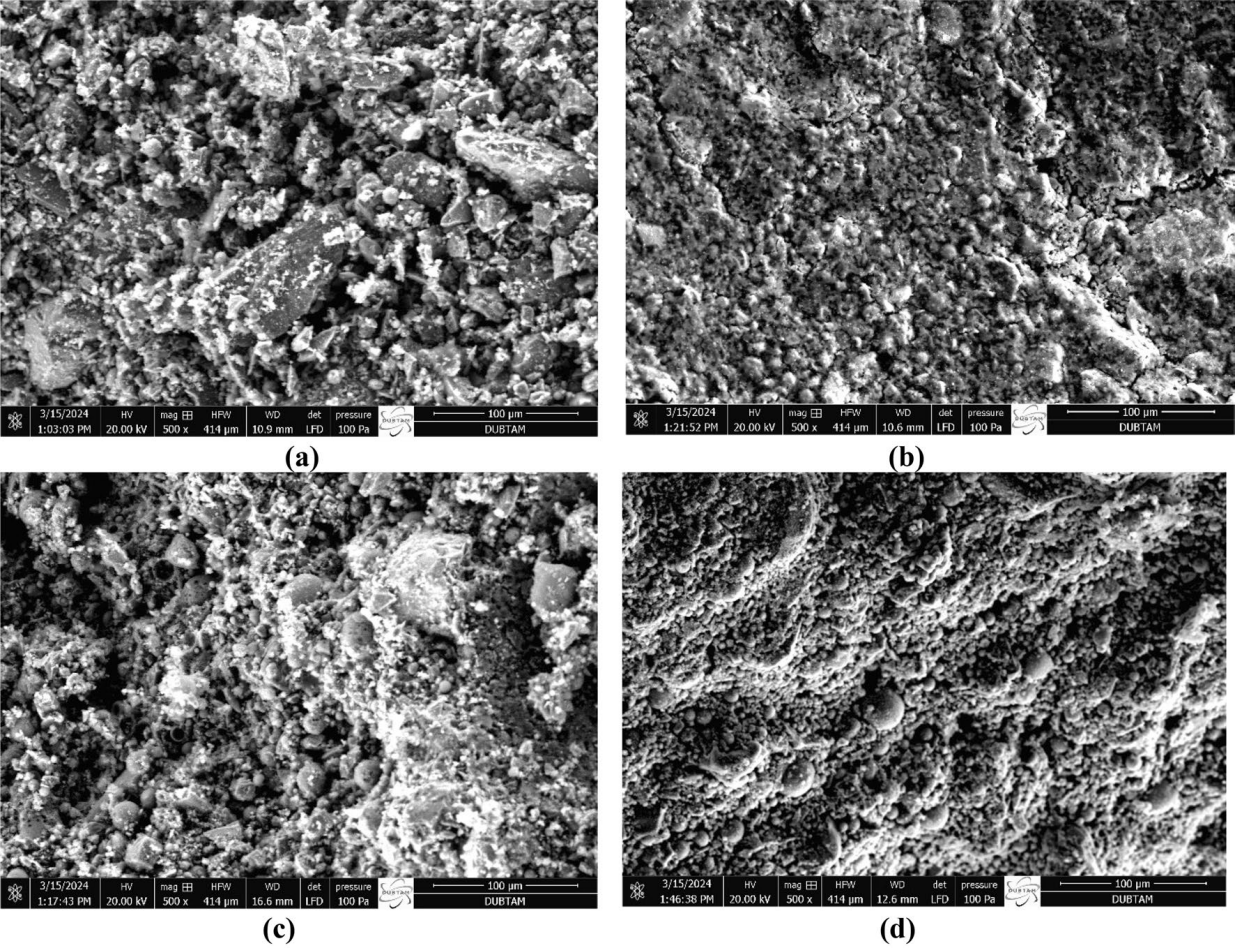
SEM images of specimens heated at 70 °C. (**a**) A1, (**b**) B4, (**c**) C1, (**d**) D4).

**Figure 23:**
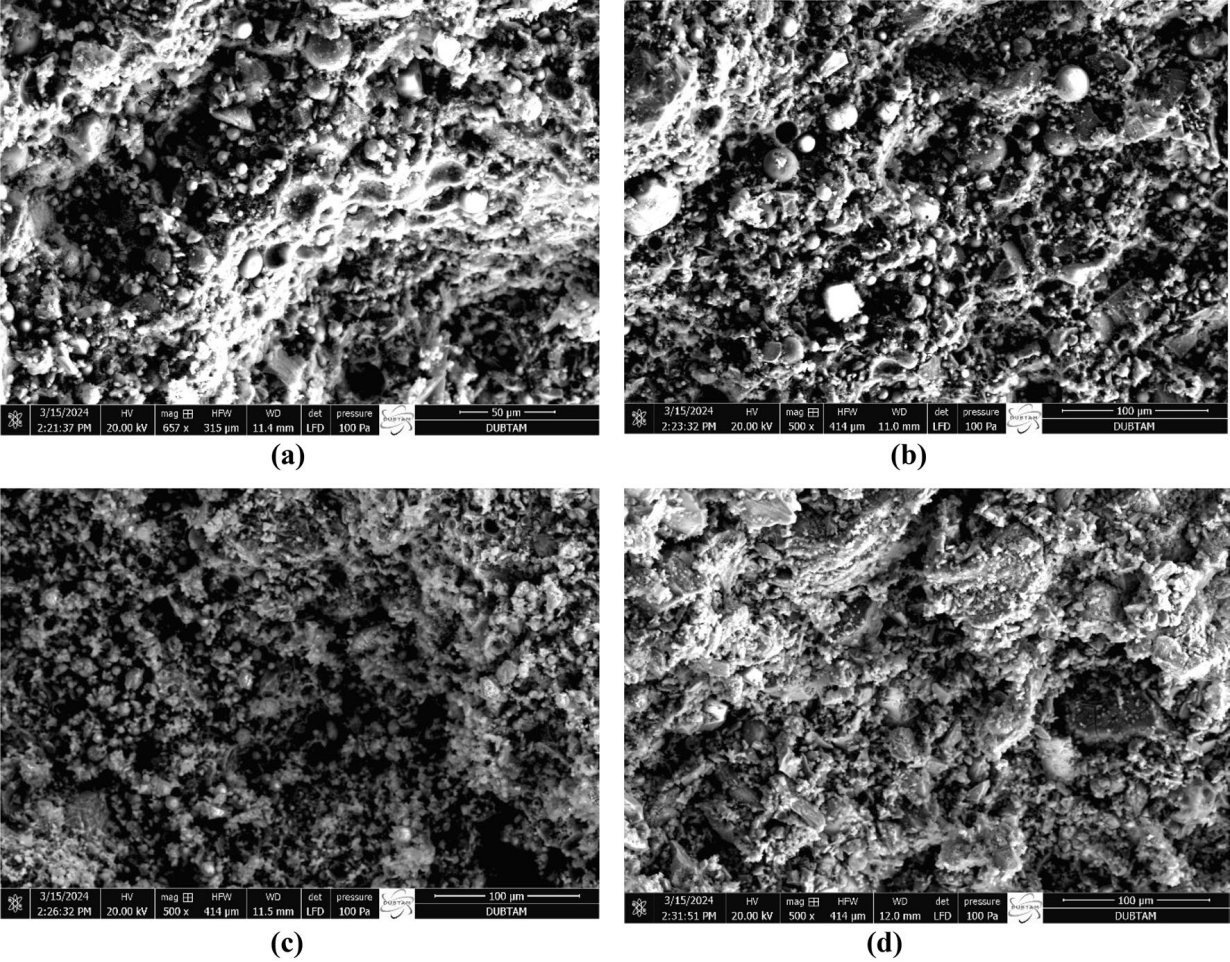
SEM images of specimens heated at 100 °C. (**a**) A5, (**b**) B8, (**c**) C5, (**d**) D8).

Figures 22 and 23 serve as visual representations illustrating the microstructures of FA-based geopolymers that underwent a 24-h heat-curing process at temperatures of 70 °C and 100 °C. These samples encompassed a spectrum of compositions, varying in FCS content (ranging from 90 to 60 wt.%) and alkali activators (including concentrations of 5 M, 10 M, as well as SS/SH ratios of 1 and 2). Upon close examination, all surfaces displayed a discernible presence of pores and voids, albeit with varying densities. This disparity in pore density correlates directly with the extent of geopolymerization and gel formation within the specimens.

A noteworthy observation is the evident reduction in density observed with diminishing FCS content, coupled with an increase in FA content in specimens subjected to curing at 100 °C. Remarkably, this trend persisted regardless of the NaOH concentration within the mixture. This microstructural phenomenon can be attributed to the observed increase in porosity values within the geopolymers, primarily driven by the dehydration of chemically bound water at temperatures surpassing 70 °C, especially in the presence of FCS. This finding aligns with the results derived from physico-mechanical properties testing, which demonstrated a clear association between higher FCS contributions and increased porosity and water absorption ratios, with decreased compressive strength and thermal conductivity values, thus providing further validation to the SEM observations.

Furthermore, the positive influence of optimal curing temperatures on the microstructure of FCS-containing geopolymers was corroborated by previous research and mechanical test outcomes. It became evident that the temperature-dependent geopolymerization reaction significantly influenced both the development of microstructure and the resultant mechanical properties, with the geopolymerization process typically being more pronounced at elevated temperatures (falling within the range of 70 to 100℃) compared to ambient conditions.

Moreover, it was established that specimens treated with NaOH 5 M exhibited a distinctly more porous microstructure when compared to their counterparts treated with 10 M, irrespective of the FCS content. This observed variation in microstructure was attributed to the higher Na/Si and Na/Al ratios inherent in the former, which are known to exert a negative influence on the geopolymerization process, a trend consistently reported in prior studies. Additionally, the density, thermal conductivity, and compressive strength values exhibited an increasing trend, while porosity and water absorption ratios showed a corresponding decrease with escalating NaOH molarities and temperature values in the mixtures. Notably, the highest and lowest compressive strength values were recorded in specimens C8 (activated with 10 M NaOH at 100 °C) and A1 (activated with 5 M NaOH at 70 °C), respectively. Noteworthy, certain specimens activated using FCS slag and FA demonstrated compressive strength values surpassing the standard threshold of 17.2 MPa, indicating their potential suitability for practical applications. Table 4 shows the elemental compositions (% by weight) of the spectral areas analyzed.

**Table 4:** EDS (wt%) of A1, B4, C1, D4, A5, B8, C5, D8 specimens.

Specimen code	Element (wt%)								
	O	Na	Mg	Al	Si	K	Ca	Cr	Fe
A1	43.80	6.49	15.39	10.03	17.48	0.27	1.55	3.14	1.86
B4	43.08	7.98	15.15	8.96	19.09	0.26	1.15	2.52	1.09
C1	44.62	12.48	12.91	9.31	15.60	0.31	1.26	2.14	1.38
D4	47.50	11.52	8.21	9.07	19.20	0.93	1.17	0.82	1.59
A5	48.81	9.05	7.00	9.59	20.50	1.03	1.33	0.76	1.95
B8	46.75	6.48	9.06	10.00	22.88	1.10	1.67	0.97	1.09
C5	44.98	7.81	13.66	10.26	17.65	0.39	1.35	2.61	1.29
D8	44.09	10.66	12.69	10.14	17.02	0.34	1.46	2.17	1.43

Finally, Electron Dispersive Spectroscopy (EDS) analysis was employed to discern the substances formed through the geopolymerization process. This analysis revealed two distinct gel formations, namely Na–Al–Si–H and Na-(Mg)–Al–Si–H. The primary hydration gel varied depending on the specimen, with some featuring sodium aluminosilicate as the dominant gel, while others showcased magnesium and sodium-magnesium aluminosilicates as the predominant forms. This variance could be traced back to the presence of magnesite in the FCS powder, which facilitates the formation of magnesium aluminosilicates. The Si/Al ratio, spanning from 1.68 to 2.29, and the Mg/Si ratio, varying between 0.34 and 0.88, were indicative of the composition and structure of the geopolymeric gels formed. However, establishing a precise correlation between these elemental ratios and the mechanical properties proved challenging due to the limited data points available for analysis by EDS.

These images, supported by the study's findings and porosity values, contribute to a comprehensive understanding of the material mechanical behavior. The microstructure of A1 was characterized by less reaction of FCS with the alkali-active solution in the geopolymer matrix. It was observed that the gel phase formed in a certain region and surrounded the fly ash grains, but in the other part of the specimen, the gel phase was not clearly formed. The resulting high porosity (18.38%) and low compressive strength (8.2 MPa) are consistent with the emphasis on semi-structural concrete. The SEM image of the specimen B4 reflects an increasing ratio of SS/SH and FA (40%) in the geopolymer matrix with decreasing porosity (11.39%), indicating a balance between increased compressive strength (17.5 MPa) and structural concrete. Higher NaOH content was observed in specimen C1. As NaOH increased, more bonding formation with silicates and aluminates led to an increase in density (2.06 g/cm3), slightly lower porosity (14.17%) than in specimen A1, which increased the compressive strength (11.12 MPa). This is consistent with the emphasis on semi-structural concrete. While examining the microstructure of specimen D4, the increase of alkaline activators in the matrix caused the geopolymer gel structure to become more homogeneous with a decreased amount of fly ash not participating in the reaction, decreased porosity (6.56%) and increased compressive strength (18.78 MPa), which is consistent with the structural concrete. SEM analysis of specimens A5, B8, C5, D8 showed that when the curing temperature was increased to 100 °C, the silica, alumina, and alkalis in the starting material, i.e. the powder binder, were dissolved for the geopolymerization reaction, then combined with each other and with other silica and alkalis in the water from the activator chemical and formed two and/or three dimensional –Si–O–Si–, –Si–O–Al–O–, –Si–O–Al–O–Al–O–Si–O– type structures. Therefore, densities decreased and porosities increased with increasing curing temperature. By increasing the curing temperature to 100 °C, the geopolymerization reaction increased and N-A-S-H crystals were produced by decomposing the unreacted fly ash, resulting in high strength. The increased porosity (20.15%) and decreased compressive strength (10.45 MPa) of the specimen A5 emphasize that it is compatible with semi-structural concrete. Both curing temperature and increasing SS/SH to FA ratio of specimen B8 emphasized that its porosity decreased (12.97%) and its compressive strength increased (19.98 MPa), which is consistent with structural concrete. It is emphasized that the gel structure increased with increasing curing temperature and NaOH content in specimen C5 and the porosity (15.48%) decreased slightly compared to specimen A5 and the compressive strength (15.02 MPa) increased, which is compatible with semi-structural concrete. In specimen D8, curing temperature, NaOH and Na2SiO3 decreased the porosity (8.81%) and increased the compressive strength (21.95 MPa), which is consistent with its emphasis on structural concrete.In summary, the SEM images visually demonstrate the tailored mixtures explored in the study, highlighting the effect of FCS, FA ratios and alkaline activators on microstructure variations. The observed trends are compatible with the study's objectives, providing a deeper understanding of the material characteristics and supporting the findings related to porosity and mechanical behavior.

## Conclusion

The structral performance characteristics of geopolymer mortars using FA and FCS activated with an alkaline solutions and cured at 70–100 °C are examined in this research. The preliminary studies determining the chemical and physical properties of FA, and FCS indicate their suitability as precursors for preparing an AAB mix.

- The results of the experimental inquiry indicated that the mixtures set and hardened in 70–100 °C were similar to OPC (ordinary portland cement). From the microstructure study (XRD, SEM and EDS) of various geopolymer specimens proportions, the presence of CSH and NASH as reaction products confirmed that the binder mixes are physically and chemically stable.
- Higher workability and slower setting time of the geopolymer mortars respectively are witnessed in the mixes with least amount of FA and least molarity of alkali solution used.
- High ratios of Na_2_O and MgO in the material may cause additional cracks in the concrete. For this reason, mixtures were prepared by determining the optimum ratios in the present study.
- It was determined that the chemical additives Na_2_SiO_3_ (sodium silicate—liquid form) and NaOH (sodium hydroxide—solid form) were used together at the specified molars and ratios to form geopolymer by reacting and gaining binding.
- The densities of the specimens produced with FCS and FA varied between 1.72 and 2.10 g/ cm^3^ and their porosities between 6.56 and 20.15%. While the lowest density value was obtained from the specimen A8, the highest porosity ratio was obtained from the specimen A5.
- It was determined that as the contribution of FCS increased, porosity and water absorption ratios increased, and compressive strength and thermal conductivity values decreased.
- The development of the microstructure and mechanical properties of the geopolymer matrix was significantly affected by the temperature-dependent geopolymerization reaction. In general, the geopolymerization reaction developed at a higher temperature (between 70 and 100 ℃) than the ambient temperature.
- Density, thermal conductivity and compressive strength values increased and porosity and water absorption ratios decreased due to the increase in NaOH molarities and temperature values in the mixtures. The highest and lowest compressive strength values were obtained in specimens C8 (10 M at 100 °C) and A1 (5 M at 70 °C), respectively. It was observed that the compressive strength values of some specimens activated using FCS and FA were above the standard value of 17.2 MPa.
- As the SS/SH ratio of geopolymer mortar specimens increased, the porosity decreased and in turn water absorption ratios decreased and thermal conductivity increased. In addition, the compressive strength of specimens with 40% FA ratio decreased with increasing SS/SH ratio. Thus, addition of more than 40% FA to the mixtures will negatively affect the mechanical properties of the specimens.
- When the thermal conductivity values were considered, the best result was obtained as 0.389 W/mK from the specimen A5 with SS/SH 1 at 100 °C curing temperature.

From this study, it can thus be concluded that the use of all AAB mixs is suitable for small scale construction work as a replacement for ordinary portland cement mix in terms of strength, durability, and sustainability. Moreover, the use of these wastes will reduce the costs of storage, minimize the level of environmental pollution by contributing to the reduction of greenhouse gas emissions, bring the available stocks into the national economy, and reduce the costs paid by enterprises for the construction of the areas where the wastes are disposed. However, the safety hazards caused by liquid sodium hydroxide during the mixing and processing of geopolymer mortar can be considered as one of the obstacles to the use of geopolymers in the concrete industry.

## Data Availability

All data generated or analysed during this study are included in this published article.
